# Characteristics of Sleep Paralysis and Its Association with Anxiety Symptoms, Perceived Stress, PTSD, and Other Variables Related to Lifestyle in Selected High Stress Exposed Professions

**DOI:** 10.3390/ijerph19137821

**Published:** 2022-06-25

**Authors:** Paulina Wróbel-Knybel, Michał Flis, Joanna Rog, Baland Jalal, Leszek Wołkowski, Hanna Karakuła-Juchnowicz

**Affiliations:** 1 I Department of Psychiatry, Psychotherapy and Early Intervention, Medical University of Lublin, Głuska 1 Street, 20-439 Lublin, Poland; michal.jerzy.pawel.flis@gmail.com (M.F.); joannarog@umlub.pl (J.R.); hannakarakulajuchnowicz@umlub.pl (H.K.-J.); 2 Department of Psychology, Harvard University, 33 Kirkland St, Cambridge, MA 02138, USA; baland_jalal@fas.harvard.edu; 3 Department of Psychiatry, University of Cambridge, Downing Street, Cambridge CB2 3EB, UK; 4 Department of Pediatric Hematology, Oncology, and Transplantology, Medical University of Lublin, Prof. Antoniego Gębali 6 Street, 20-093 Lublin, Poland; leszek.wolkowski@gmail.com

**Keywords:** sleep paralysis, anxiety, parasomnia, sleep disorder, PTSD, health status

## Abstract

Sleep paralysis (SP) is a hypnagogic or hypnopompic state associated with the inability to move while conscious. Recurrent isolated sleep paralysis (RISP) is a type of REM parasomnia. Individuals experiencing anxiety disorders, PTSD, exposure to chronic stress, or shift work are at risk of developing this sleep disorder. This study aimed to assess: (1) the prevalence, frequency, and symptomatology of SP, and (2) the impact of the severity of anxiety symptoms, perceived stress, and lifestyle mode variables on the frequency and severity of SP in four professional groups at high risk of SP (*n* = 844): nurses and midwives (*n* = 172), policemen (*n* = 174), teachers (*n* = 107), and a group of mixed professions—“other professions” (*n* = 391). The study used a battery of online questionnaires: the Sociodemographic and Health Status Questionnaire, the SP-EPQ, the PCL -5, the STAI-T, the PSWQ and the PSS-10. The prevalence of SP was the lowest among policemen (15.5%) and the highest in the group of “other professions” (39.4%). The association of SP with symptoms of PTSD and anxiety was confirmed in the group of nurses and “other professions”. Among other factors modulating the incidence and severity of SP were: age, BMI, smoking, alcohol consumption, sleep duration, and perceived stress. This study indicates that there exist links between SP and psychological and lifestyle factors, suggesting a complex etiology for this sleep disorder. Due to the high prevalence of SP in the studied groups of occupations, further research is necessary to develop preventive and therapeutic methods for SP.

## 1. Introduction

Sleep paralysis (SP) is a transitional dissociative state that usually occurs while waking up from sleep or falling asleep [1]. SP episodes manifest as an inability to move (muscle atonia) as in normal sleep, but the person stays awake [1,2]. It often proceeds with hypnopompic and hypnogogic hallucinations: visual, tactile, kinesthetic, auditory, and less often olfactory [3,4,5]. There are three types of hallucinations typical of SP: (1) the “intruder”, associated with a sense of a threatening/hostile presence, fear, auditory and visual hallucinations (often a shadow or dark form); (2) the “incubus”, associated with a feeling of tightness or pain on the chest, breathing difficulties, and sometimes visual hallucinations (figure sitting on the chest); and (3) vestibular-motor hallucinations, described as unusual bodily sensations such as feelings of levitation, spinning, autoscopy, and out-of-body experiences (OBEs) [3]. SP episodes are often accompanied by various psychosomatic sensations, among which are the occurrence of the feeling of pressure on the chest, difficulty breathing, a feeling of suffocation, heart palpitations, sweating, and nausea [5]. This is usually associated with increased fear or fear of dying [5,6].

There are several theories about the pathophysiology of this disorder, but none are fully confirmed. Among others, it is considered to be associated with an abnormal overlapping of REM sleep with the waking state [7]. In polysomnographic studies, abnormalities such as fragmentation of the REM sleep phase and shorter REM sleep latencies than normal along with shortened NREM and REM sleep cycles were observed in patients experiencing SP [8].

SP occurs in isolation, known as isolated sleep paralysis (ISP), or as a symptom of other medical conditions, e.g., narcolepsy, seizure disorders [1]. Repeated episodes of ISP are considered as a specific diagnostic sleep-wake disorder called recurrent isolated sleep paralysis (RISP), which is a type of REM sleep phase parasomnia.

The International Classification of Diseases (ICD-11) in the group of parasomnias related to the REM sleep phase under the code 7B01.1 distinguishes recurrent isolated sleep paralysis (RISP), defining it as “consisting of the recurrent inability to move the trunk and all of the limbs at sleep onset (hypnagogic) or upon awakening (hypnopompic) from sleep. Episodes typically last from a few seconds to a few minutes and cause clinically significant distress including bedtime anxiety or fear of sleep.”. RISP was not coded in DSM-5, but it can be marked as 307.40 (unspecified sleep-wake disorder) or 307.49 (other specified sleep-wake disorder) [9].

The prevalence of ISP varies depending on cultural and social factors and health status [10,11]. The results of the research also revealed that lifestyle variables, sleep hygiene, stress, and genetic susceptibility may be associated with its more frequent occurrence [12,13,14] and intensity [5]. A 2011 literature review of 35 aggregated studies indicated that approximately 8% of the general population had experienced at least one episode of SP at some point in their life, as did 28% of students, and 32% of psychiatric patients [10]. The most common mental disorders with which SP coexists are post-traumatic stress disorder (PTSD), panic disorder, social phobia, and generalized anxiety disorder [14]. Studies show that from 27.8% to 76% of patients diagnosed with PTSD experienced at least one sleep paralysis in their lifetime [14].

### Occupations Exposed to Stress and at an Increased Risk of SP

In the general population, one of the most common sleep disorders is insomnia, and its prevalence is estimated to range between 6 and 15%, while poor-quality sleep is experienced by up to 30% of people around the world [15]. Among the factors that negatively affect sleep quality and may cause sleep disorders is acute and chronic stress [15,16]. We suppose that the prevalence of SP, as with other sleep disorders, may be higher in occupational groups particularly exposed to stress. Unfortunately, no studies have compared SP and other sleep disorders among different occupational groups. However, based on scientific articles, we find that healthcare professionals working in shifts, policemen, and firefighters are more likely to experience sleep problems [15,17,18,19,20,21,22]. These professional groups belong to occupations at high risk of stress [23,24,25,26,27,28]. They are burdened with greater exposure to stress and high social pressure due to their functions, and thus an increased risk of developing anxiety and sleep disorders [17,18,19,23,24,25,26,27,28]. According to a meta-analysis from 2019, on average, 51% of police officers complained of experiencing poor sleep quality [20]. Another study of 4597 North American police officers found that as many as 40.4% have a sleep disorder [17]. Sleep disorders afflict as many as 40.9% of health care workers, wherein 19% of nurses have a positive diagnosis of insomnia [19]. In a study conducted in Poland, 48% of nurses reported symptoms indicating insomnia [21]. Even though teachers do not work at night or perform work that triggers acute stress compared to other professions, teaching is considered to be one of the most stressful professions, due to chronic stress (59–100%) [29,30]. In this professional group, exposure to occupational stress is associated with the occurrence of anxiety (67.5%) and symptoms of depression (23.2%) [30]. Teaching is also related to a high risk of occupational burnout [31,32], and many teachers decide to quit their job—17% after working for a year and 10% after at least 10 years of experience [33]. There are few studies on sleep quality among teachers, but according to them, poor sleep quality is experienced by 46–51% of teachers [29,34]. Additionally, nurses, midwives, and policemen are burdened with the consequences of working shifts, which additionally increases the risk of depression, anxiety, and sleep disorders [17,19,20,35,36]. These occupational groups are also exposed to extreme situations related to the performance of their work and thus are at an increased risk of PTSD [23,24,25,26], which is one of the risk factors for SP. Increased stress load due to factors such as late or irregular shifts, exposure to acute and chronic stress, a higher risk of sleep disorders, anxiety disorders, and a higher PTSD burden among these occupations suggests that they may be at risk for SP. We suppose that the prevalence of sleep paralysis in these professional groups may be higher than in the general population, and is related to the burden associated with the nature of the work performed.

The aim of the study is to investigate (I) the prevalence, frequency, and symptomatology of SP; (II) the impact of anxiety symptoms, perceived stress, and lifestyle mode variables on the frequency and severity of SP in the adult Polish population, based on four high-stress-exposed professional groups at high risk of SP: (1) nurses and midwives; (2) teachers; (3) policemen and (4) a group of mixed professions—“other professions”.

## 2. Measures and Methods

### 2.1. Participants and Procedure

This study was based on a battery of online questionnaires (constructed and distributed by the www.survio.com platform, access date: 21 April 2022) and was carried out in four groups of respondents, each cohort differing in their professional work: (1) nurses and midwives, (2) teachers, (3) policemen, and (4) “other professions”.

(1) Nurses and midwives: recruited through advertisements on newsgroups and forums pertaining to these professions more generally. The questionnaire was completed by 172 participants (170 women, 2 men).
(2) Teachers: recruited via an e-mail invitational study sent to schools. The questionnaire was completed by 108 participants (98 women, 10 men).
(3) Policemen: recruited after obtaining consent from the Commander of the Main Headquarters of Police. The research questionnaire was made available to police officers by email to 40 police units located all over Poland. Online research surveys were available by e-mail to policemen working in these units. The questionnaire was completed by 174 participants (49 women, 125 men).
(4) “Other professions”: a battery of questionnaires was made available on the https://www.facebook.com/paralizsenny/website (access date: 21 April 2022) from March 2019 to November 2019. The questionnaire was completed by 391 participants (285 women, 106 men). A list of professions included in the group “other professions” is presented in Supplementary Table S1.

**Figure 1:**
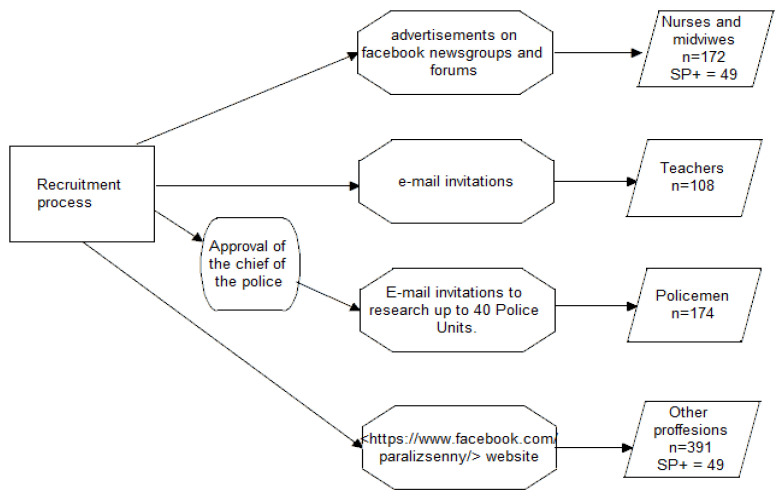
Study participants and procedure: flowchart of the procedure of recruiting participants to study.

The procedure of recruiting participants to the study is shown in Figure 1.

All of the data were collected from March 2019 to November 2019. In total, the survey was completed by 844 adult responders. All potential subjects were presented with a consent form and were not allowed to proceed until they acknowledged that they: (1) had read the information about the study, (2) were 18 years of age or older, and (3) had read and understood the consent form specifying that participation is voluntary and the results are anonymous. Furthermore, all participants completed a battery of online self-rating questionnaires: (1) a personal questionnaire, (2) the Sleep Paralysis Experiences and Phenomenology Questionnaire (SP-EPQ) [37], (3) The PTSD Checklist (PCL) [38], (4) The State-Trait Anxiety Inventory (STAI-T) [39], and (5) The Penn State Worry Questionnaire (PSWQ) [40], The Perceived Stress Scale (PSS-10) [41].

### 2.2. Measures

#### 2.2.1. Sociodemographic and Health Status Questionnaire

The questions included in the Sociodemographic and Health Status Questionnaire concerned:

I. personal data, i.e., gender, age, height, weight, size of the city in which they live, profession, and working time (permanent/shift)
II. lifestyle data, i.e., smoking (number of cigarettes smoked during the day and smoking time in years), the average number of hours of sleep during the night, alcohol consumption (type of alcohol and frequency of drinking), the number of cups of coffee or other caffeinated beverages consumed during the day, and physical activity (number of hours a week spent in physical activity).
III. health data, i.e., the presence of chronic diseases (somatic and psychiatric) and medications taken.

#### 2.2.2. Sleep Paralysis Experiences and Phenomenology Questionnaire

The Sleep Paralysis Experiences and Phenomenology Questionnaire (SP-EPQ) is a tool for assessing the frequency, characteristics (e.g., symptomatology, episode circumstances) of sleep paralysis episodes, and the level of knowledge about the experience [37]. The questionnaire has been used in Italy, Turkey, and Poland [5,11,42,43].

The SP-EPQ consists of twelve open-ended and five closed-ended questions regarding SP. The first question of the survey was as follows: “Some people experienced an event in which they could not move their arms, legs or speak during sleep or waking up, even though they wanted to do it: Have you ever had such an experience?”. If participants answered yes to this question, they were asked to describe the episode, thus making it possible to verify that the experience was in fact SP. The other eleven questions from the questionnaire were formulated in the same way—requiring users to describe the reported experiences—with the exception being point 8 of the questionnaire, which consisted of twelve closed-ended questions regarding the presence of somatic SP symptoms [37]. The Cronbach’s alpha coefficient for this part of the test in our study was 0.71.

#### 2.2.3. The PTSD Checklist (PCL-5)

The PTSD Checklist (PCL-5) used in this study is the Polish version of the PCL questionnaire developed for the DSM-5 [38,44]. The tool consists of 20 items and assesses the severity of PTSD symptoms on a 5-point scale from 0 (not at all) to 4 (extremely) [44]. The Cronbach’s alpha coefficient for this study was 0.92.

#### 2.2.4. The State-Trait Anxiety Inventory (STAI)

In the study, we used subscale X-2, the Polish version of the STAI questionnaire [39,45]. The subscale consists of 20 questions. It is used to assess anxiety as a personality component (trait). On a 5-point Likert scale, the respondents mark to what extent the behavior described by the statement is typical for them [39]. The Cronbach’s alpha coefficient for this study was 0.88.

#### 2.2.5. The Penn State Worry Questionnaire (PSWQ)

In our study, we employed a Polish adaptation of the PSWQ Questionnaire consisting of 16 statements describing various manifestations of worry [40,46]. The questionnaire is based a 5-point Likert scale, where participants mark to what extent the behavior described is typical for them. Responses range from 1 (not at all typical for me) to 5 (very typical for me). The theoretical maximum score is 80, while the minimum is 16, where a higher score indicates a greater tendency to worry [40]. The Cronbach’s alpha coefficient for this study was 0.94.

#### 2.2.6. The Perceived Stress Scale (PSS-10)

A Polish adaptation of the PSS-10 scale [41,47] was used to measure the perceived stress over the last month. It contains 10 questions about subjective feelings concerning different problems, stress-related events, and ways of coping with stress. The tool is characterized by good psychometric properties [41]. The Cronbach’s alpha coefficient calculated for our study was 0.77.

### 2.3. Statistical Analysis

Statistical analysis was performed using Statistica software (TIBCO Software Inc., 100 Palo Alto, CA, USA). Continuous variables were expressed in terms of means, medians, and qualitative variables in numbers and percentages. Due to the non-Gaussian distribution of continuous variables, non-parametric tests were applied to determine the relations and differences. To examine the null hypothesis, the Mann–Whitney test was used. We used the Kruskal–Wallis test to examine differences between examined variables in the subgroups. A chi-square test was applied to find differences in categorical variables. We used the rho-Spearman rank correlation test to determine the relationship between continuous variables. Cronbach’s alpha coefficient was calculated for reliability analysis of psychological scales and subscale assessments of psychosomatic symptoms related to SP. The effect size was calculated using epsilon squared for the Kruskal–Wallis H test using following formula, $\frac{X}{\left(n^{2}-1\right)\left(n+1\right)}$, and using eta squared for the Mann–Whitney U test: Z^2^/(*n*−1). Effect size measures the degree to which the phenomenon is present in the population. Recommended minimum effect size representing a “practically” significant effect for science data (RMPE) was established at ε2 and ɳ2 = 0.04 [48]. In an analysis of two groups, a p-value of less than 0.05 was considered statistically significant. In cases of multiple comparisons, the p-value was adjusted by applying the Bonferroni correction.

## 3. Results

### 3.1. Characteristics of the Study Group

#### 3.1.1. The Demographic Characteristics, Lifestyle, and Health Status of the Study Participants

In total, 844 participants between 18 and 67 years of age participated in this study. The sociodemographic characteristics of the study group by profession are presented in Table 1.

The current health status of participants by profession is presented in Table 2.

#### 3.1.2. Differences between Professions in the Study Group (SP+ and SP−)

Statistically significant differences between the studied professional groups concerned: age, BMI, coffee and alcohol consumption, sleep duration, time spent on physical activity, self-assessed scales (STAI, PCL-5, PSWQ). Table 3 contains characteristics of the study group by the professions in terms of the variables of lifestyle. The number of points in the self-report scales, STAI, PCL-5, PSWQ, and PSS-10, in each study group is presented in Table 4

##### Age Differences

Participants from the “other professions” cohort were significantly younger than nurses and midwives (H = 5.22; *p* = 0.000001), teachers (H = 6.64; *p* = 0.000001) and policemen (H = 10.27; *p* = 0.000001). Nurses and midwives were significantly younger than policemen (H = 4.27; *p* = 0.0001). The effect size for age differences was ε2 = 0.15.

##### Differences in Body Mass Index (BMI)

The policemen cohort had significantly higher BMI than nurses and midwives (H = 4.24; *p* = 0.0001), teachers (H = 4.55; *p* = 0.00003) and the “other professions” group (H = 4.14; *p* = 0.0002). The effect size for BMI differences was ε2 = 0.061.

##### Differences in Alcohol Consumption

Police officers consumed alcohol more often compared to nurses and midwives (H = 4.66; *p* = 0.00002) and “other professions” (H = 2.91; *p* = 0.02). The effect size for alcohol consumption was ε2 = 0.029 (below RMPE).

##### Differences in Sleep Duration

The participants from the “other professions” group on average slept significantly longer during the night than nurses and midwives (H = 3.93; *p* = 0.0005) and policemen (H = 4.75; *p* = 0.0001). The average sleep duration of policemen was significantly shorter than that of teachers (H = 2.68, *p* = 0.04). The effect size for differences in sleep duration was on the verge of RMPE: ε2 = 0.038. There were no significant differences in the average number of hours of sleep reported by teachers and “other professions”.

##### Differences in Time Spent on Physical Activity

The policemen group, on average, spent more time on physical activity as compared to each of the other groups: nurses and midwives (H = 4.33; *p* = 0.00009), teachers (H = 5.37; *p* = 0.00001), and “other professions” (H = 2.96, *p* = 0.02) with effect size ε2 = 0.056.

##### Differences in the results of self-written scales (STAI, PCL-5, PSWQ)

The policemen group had significantly lower score in the STAI questionnaire than each of the other studied groups: nurses and midwives (H = 4.63; *p* = 0.00002), teachers (H = 3.95; *p* = 0.0005) and the “other professions” cohort (H = 3.29; *p* = 0.006).

Nurses and midwives had significantly more PTSD symptoms reported in the PCL-5 questionnaire than “other professions” (H = 2.91; *p* = 0.02).

Police officers had a significantly lower score in the PSWQ questionnaire than the other three groups: nurses and midwives (H = 5.34; *p* = 0.00002), teachers (5.48; *p* = 0.0005) and “other professions” (H = 4.08; *p* = 0.006). Teachers had a higher PSWQ score than “other professions” (H = 2.76; *p* = 0.034).

Police officers had a significantly lower score in the PSS-10 questionnaire than the group of nurses and midwives (H = 2.66; *p* = 0.05) and teachers (H = 2.97; *p* = 0.02).

The effect size for STAI was ε2 = 0.031 (below RMPE), for PCL-5 it was ε2 = 0.014 (below RMPE), for PSWQ it was ε2 = 0.048 (higher than RMPE), and for PSS-10 it was ε2 = 0.013 (below RMPE).

#### 3.1.3. Differences between Professions in the Group of Participants Who Experienced at Least One Episode of SP (SP+)

Statistically significant differences between the studied professional groups (SP+) concerned: age, BMI, sleep duration, time spent on physical activity.

##### Age Differences

In the group of SP+ participants, the differences in the metric age were statistically significant and the effect size was ε2 = 0.17. The group of “other professions” was significantly younger than nurses and midwives (H = 5.22; *p* = 0.000001), teachers (H = 3.90; *p* = 0.0006) and policemen (H = 2.74; *p* = 0.036). The difference in age was also significant between nurses and policemen (H = 3.09; *p* = 0.012).

##### Differences in Body Mass Index (BMI)

The group of policemen had significantly higher BMI than teachers (H = 2.69; *p* = 0.043), nurses and midwives (H = 3.46; *p* = 0.0033), and “other professions” (H = 4.28; *p* = 0.0001). The effect size for BMI differences was ε2 = 0.073.

##### Differences in Sleep Duration

Among SP+ participants, a significant difference in the number of hours of sleep per night was apparent between policemen and nurses and midwives and “other professions”. SP+ policemen, on average, slept significantly less during the night than nurses and midwives (H = 2.90; *p* = 0.022) and “other professions” (H = 4.29; *p* = 0.0001). The effect size for the duration of sleep was ε2 = 0.083.

##### Differences in Time Spent on Physical Activity

In the SP+ group, police officers on average spent more time on physical activity on average compared to nurses and midwives (H = 2.92; *p* = 0.021), with an effect size ε2 = 0.046.

### 3.2. Prevalence and Characteristics of SP

#### 3.2.1. Lifetime and Period Prevalence of SP

At least one episode of SP was experienced by 27.91% of nurses and midwives (*n* = 48), 26.17% of teachers (*n* = 28), 15.52% of policemen (*n* = 27), and 39.39% of representatives of the “other professions” group (*n* = 154).

The number of SP episodes in the last month, year, and throughout the participant’s lifetime did not differ significantly between study groups of professions. The frequency distribution of SP episodes is presented in Table 5.

#### 3.2.2. Characteristics of SP Episodes

The SP episode characteristics of each of the studied professional cohorts are presented in Table 5. The factors characterizing SP, presented in Table 5, did not differ significantly between occupational groups.

#### 3.2.3. Symptomatology of SP

The psychosomatic symptoms experienced by the participants are presented in Table 6, along with their prevalence in each of the studied professional cohorts. The policemen group reported fewer SP-associated psychosomatic symptoms when compared with the “other professions” group (H = 4.67; *p* = 0.00002; ε2 = 0.043).

Table 7 presents the prevalence of hallucinations depending on the sensual modality; feelings of derealization; depersonalization; fear; and fear of death reported during SP in each of the studied cohorts.

Responders from the “other professions” group reported more hallucinations during SP compared with teachers (H = 2.72; *p* = 0.0003) and policemen (H = 2.72; *p* = 0.039), with an effect size ε2 = 0.043. Participants from the “other professions” group also reported more symptoms of SP in general (listed in Table 6 and Table 7) when compared to policemen (H = 4.88; *p* = 0.000006), with an effect size ε2 = 0.046. There were no other significant differences in the quality and quantity of SP symptoms between professions.

### 3.3. Relationship between SP and Anxiety Symptoms and Perceived Stress

#### 3.3.1. The Association between Frequency of SP Episodes and Trait Anxiety Symptoms (STAI-T)

In the group of “other professions”, a positive correlation was found between the number of points in the STAI-T questionnaire and the number of SP episodes during the last year (R = 0.17; *p* < 0.05).

Among nurses and midwives, there was a positive correlation between the number of points in the STAI-T questionnaire and the number of SP episodes during the last month (R = 0.29; *p* < 0.05) and lifetime episodes (R = 0.30; *p* < 0.05).

In the remaining researched professional groups, there was no relationship between the number of STAI-T points and the number of SP episodes.

#### 3.3.2. The Relationship between the Number of SP Symptoms Reported and Trait Anxiety Symptoms (STAI-T)

Among all respondents, only within the “other professions” group was there found to be a positive correlation between the number of points in the STAI-T questionnaire and the number of reported hallucinations (R = 0.23; *p* < 0.05), psychosomatic symptoms (R = 0.19; *p* < 0.05) and the total number of all reported SP symptoms (R = 0.27; *p* < 0.05).

#### 3.3.3. The Association between Frequency of SP Episodes and Severity of Symptoms of Post-Traumatic Stress Disorder (PTSD)

In the “other professions” group, there was a positive correlation between the intensity of PTSD symptoms in the PCL-5 questionnaire and the number of SP episodes in the last month (R = 0.27; *p* < 0.05), year (R = 0.32; *p* < 0.0.5) and total lifetime (R = 0.18; *p* < 0.05).

In the other examined professional cohorts, there was no correlation between the number of SP episodes in the measured time intervals and the intensity of PTSD symptoms on the PCL-5 scale.

#### 3.3.4. Relationship between the Number of SP Symptoms Reported and the Severity of Symptoms of Post-Traumatic Stress Disorder (PTSD)

In the nurses and midwives group, there was a positive correlation between the number of points in the PCL-5 questionnaire and the number of reported psychosomatic symptoms (R = 0.33; *p* < 0.05), as well as all SP symptoms (R = 0.32; *p* < 0.05).

In the “other professions” group, there was also a positive correlation between the intensity of PTSD symptoms measured by PCL-5 and the number of reported SP symptoms: psychosomatic symptoms (R = 0.23; *p* < 0.05), hallucinations (R = 0.17; *p* < 0.05), and all reported SP symptoms (R = 0.26: *p* < 0.05).

There was no correlation between the intensity of PTSD symptoms measured on the PCL-5 scale and the number of reported SP symptoms in any of the studied professional groups.

#### 3.3.5. SP and Tendency to Worry (PSWQ)

In the “other professions” group, there was a positive correlation between the number of points in the PSWQ questionnaire and the number of all reported symptoms during SP (R = 0.19; *p* < 0.05).

#### 3.3.6. SP and Intensity of Perceived Stress (PSS-10)

In the nurses and midwives group, there was a positive correlation between the intensity of perceived stress on the PSS-10 scale and the number of reported SP episodes during the last month (R = 0.31; *p* < 0.05) and past year (R = 0.31; *p* < 0.05).

In the teachers group, teachers who had experienced at least one episode of SP within their lifetimes scored higher on the PSS-10 scale compared to those who never experienced SP, M = 23.64, Me = 24.0, range = 28.0 vs. M = 20.61, Me = 20.0, range = 28.0 (Z = 2.17; *p* = 0.03; ɳ2 = 0.17).

### 3.4. Influence of Age, BMI, and Lifestyle Variables on the Frequency and Severity of SP Episodes

This section describes the significant results regarding the impact of the following factors on SP: age, body mass index (BMI), smoking (number of cigarettes per day, duration of nicotine dependence, alcohol and coffee consumption, physical activity and sleep duration.

The results for each professional group are described in a separate sub-section.

#### 3.4.1. Nurses and Midwives

Nurses and midwives who had experienced at least one episode of SP ever were on average younger than those who never experienced SP, M = 34.04, Me = 30.5, range = 34.0 vs. M = 38.33, Me = 39.0, range = 41.0 (Z = 2.14; *p* = 0.03).

The number of SP episodes over the last year was negatively correlated with age (R = 0.39; *p* < 0.05) and was also negatively correlated with the mean number of caffeinated beverages consumed during the day (R = −0.34; *p* < 0.05).

Nurses and midwives who had experienced at least one episode of SP ever had a lower BMI than those who never experienced SP, M = 24.11, Me = 23.05, range = 23.05 vs. M = 25.86, Me = 24.67, range = 24.26 (Z = 2.3; *p* = 0.02; ɳ2 = 0.027—below RMPE).

In this group, a negative correlation was found between BMI and the number of reported psychosomatic symptoms of SP (R = 0.41; *p* < 0.05) and all symptoms of SP (R = 0.42; *p* < 0.05).

Participants who experienced at least one episode of SP, on average, slept more than those who never experienced SP, M = 7.06, Me = 7.0, range = 5.0 vs. M = 6.6, Me = 6.5, range = 6.0 (Z = 2.11; *p* = 0.04; ɳ2 = 0.031) However, the effect size was below RMPE.

#### 3.4.2. Teachers

In the group of teachers, there was a positive correlation between the number of reported SP symptoms and BMI (R = 0.42; *p* < 0.05). A negative correlation was found between the number of reported psychosomatic symptoms and the number of caffeinated beverages consumed (R =−0.38; *p* < 0.05) and average sleep duration (R =−0.39; *p* < 0.05).

#### 3.4.3. Police Officers

A negative correlation was found to exist between the age of the surveyed policemen and the number of SP during the last month (R =−0.46; *p* < 0.05) and year (R =−0.45; *p* < 0.05).

In the policemen group, there was a positive correlation between the number of cigarettes smoked per day and the number of reported psychosomatic symptoms of SP (R = 0.44; *p* < 0.05), and the number of all reported symptoms of SP (R = 0.41; *p* < 0.05). There was also a positive correlation between the number of years of cigarette smoking and the number of reported psychosomatic symptoms of SP (R = 0.42; *p* < 0.05) and all reported symptoms (R = 0.41; *p* < 0.05).

Participants in the policemen cohort who had experienced at least one episode of SP in their lifetime, on average, drank fewer cups of coffee per day than participants who had never experienced SP, M = 1.2, Me = 1.0, range = 3.0 vs. M = 2.13, Me = 2.0, range = 14.0 (Z = 2.08; *p* = 0.005; ɳ2 = 0.025). This result was also below RMPE.

There is a positive correlation between the frequency of alcohol consumption and the number of reported SP symptoms (R = 0.39: *p* < 0.05)

Policemen who had experienced at least one episode of SP, on average, slept less than those who had never experienced SP, M = 6.15, Me = 6.0, range = 4.0 vs. M = 6.68, Me = 7.0, range = 5.0 (Z = 2.28; *p* = 0.02; ɳ2 = 0.03—below RMPE).

#### 3.4.4. “Other Professions”

In the “other professions” cohort, there was found to be a negative correlation between the age of the respondents and the number of SP episodes during the last year (R = −0.22; *p* < 0.05) and the number of psychosomatic symptoms reported during SP (R = −0.18; *p* < 0.05).

In this group, participants who had experienced at least one episode of SP ever were on average younger than those who had never experienced SP, M = 29.34, Me = 27.5, range = 42.0 vs. M = 34.28, Me = 32.0, range = 65.0 (Z = 4.45; *p* = 0.000009; ɳ2 = 0.05).

Participants who had experienced at least one episode of SP ever had a lower BMI than those who had never experienced SP M = 24.04, Me = 23.04, range = 36.38 vs. M = 25.28, Me = 23.94, range = 41.79 (Z = 2.13; *p* = 0.03; ɳ2 = 0,012). Nevertheless, both of these results were below RMPE.

There was a positive correlation between the number of cigarettes smoked during the day and the number of SP episodes in the last month (R = 0.24; *p* < 0.05), year (R = 0.19; *p* < 0.05) and the number of hallucinations reported during SP (R = 0.20; *p* < 0.05) and all symptoms (R = 0.20; *p* < 0.05).

There was also a positive correlation between the duration of smoking in years and the number of SP episodes in the last month (R = 0.23; *p* < 0.05), the number of hallucinations reported (R = 0.20; *p* < 0.05), and all symptoms during SP (R = 0.20; *p* < 0.05).

## 4. Discussion

The aim of this study was to assess the prevalence of SP in three selected high-stress-exposed professional groups (policemen, nurses and midwives, and teachers) burdened with a high risk of sleep and anxiety disorders, which has been realized. The other objectives of the study were also achieved, i.e., performing a comparison of the symptomatology, intensity, and frequency of SP in selected professional cohorts: nurses and midwives, teachers, policemen and a group of professionally active people in various industries (“other professions”), wherein the impact of anxiety symptoms, perceived stress and other factors on the occurrence and severity of SP was examined.

In our study, the highest lifetime prevalence of SP was in the “other professions” group—namely, 39.39%. However, these findings should be interpreted cautiously, as the questionnaire was placed on a website about SP, and therefore was likely filled out by people who were particularly interested in the subject of SP. Due to this, we can conclude that this site was visited more often by people suffering from SP. Therefore, we will not discuss prevalence in the “other professions” group, nor compare its prevalence with that of other professional groups examined by us.

In each of the professional groups, lifetime prevalence was higher than the average for the general population—7.6% [10]. The course of SP episodes in the groups we studied was typical, as already described by other researchers [3,5,49,50]. Study participants reported the presence of hypnopompic and hypnagogic hallucinations of various modalities. They also described hallucinations characteristic of SP, as described by Cheyne et al.: intruder, incubus and vestibular-motor [3]. The types of hallucinations reported did not differ significantly between studied groups. The number of reported symptoms was the highest in the “other professions” group and the lowest in the policemen group. The frequency of SP episodes did not differ between the professional groups we studied.

We shall start by discussing the prevalence of SP in the policemen cohort and the variables influencing the frequency and severity of SP within this group. The prevalence rate in the policemen cohort was the lowest among all of the occupational groups examined by us—15.52%. However, the prevalence in this group was higher than in the population of Polish firefighters tested by our team in another survey, which was 8.7% [11]. It is worth mentioning that in the group of policemen examined by us, the intensity of anxiety symptoms on the STAI and PSWQ scales and the intensity of perceived stress on the PSS-10 scale were statistically lower than in other professional groups we studied. Of note, the intensity of PTSD symptoms measured in the PCL-5 questionnaire was higher in policemen than in the “other professions” cohort.

These results coincide with the observations of other researchers that police officers are a professional group characterized by high mental resilience, with their mental reactions to traumatic events being milder than those of the civilian population [24,25]. This, however, does not change the fact that due to the higher-than-average occupational exposure to traumatic situations, they are at risk of PTSD, depression, and anxiety disorders development [23,51]. Interestingly, when only comparing the participants of the study who experienced at least one episode of SP, there are no significant differences in the scores on the scales assessing the severity of anxiety symptoms used in our study. This might indicate that policemen who experience SP have similar levels of anxiety and PTSD symptoms as civilians who have experienced at least one SP, including also other occupations exposed to above-average occupational stress such as nurses. Whether SP is a response to high stress and whether its occurrence is determined by the individual’s predisposition to react with anxiety (STAI, PSWQ) are questions that remain open for further research on adaptive possibilities and mental resilience.

In the policemen cohort, the number and severity of SP episodes was in no way related to the results of the self-description scales we used (STAI, PSWQ, PSS-10, PCL-5). In this group, the importance was the association of SP with age. We found a negative correlation between the age of the respondents and SP episodes during the last month and last year. The same relationship was also noticed in the nurses and midwives cohort as well as in the other professions cohort. Currently, no previous studies confirm that the incidence of SP is age-related [14]. However, studies conducted in student populations indicate that the lifetime prevalence of SP is higher (27.8%) than in the general population [10]. This is in line with the results of our research which show that the age of the respondents was negatively correlated with the number of episodes of SP in the last month and year (but not the entire lifetime) [14].

Among policemen, a positive correlation was found between the number of cigarettes smoked and number of years smoking and reported psychosomatic symptoms of SP. Additionally, alcohol consumption in this group was positively correlated with the sum of all reported symptoms of SP. Similarly, in the group of “other professions”, both the number of cigarettes smoked and the duration of smoking were related to the frequency and intensity of SP. There is not much research into the effects of nicotine on sleep. However, their results confirm that smokers experience REM sleep latency, a decrease in slow-wave sleep, an increase in α-frequencies, and a reduction in δ-frequencies compared to non-smokers [52]. In addition, there are reports that smoking may also be associated with SP.

In the study conducted in a group of Polish students, there was a positive correlation between the number of cigarettes smoked and the number of psychosomatic symptoms of SP [5]. Munezawa et al. also noted the association between alcohol and nicotine and SP in their study. The results of the study conducted in a group of teenagers indicate a higher incidence of SP among alcohol users (7.1% vs. 12.2%) and cigarette smokers (7.8% vs. 15.3%) compared to those who did not use these psychostimulants [53].

Although there was no relationship between SP and alcohol in any of the other occupational cohorts we studied, it seems interesting to examine the relationship between alcohol and SP in a group of policemen and Japanese teenagers examined by Munezawa et al. The negative effect of alcohol on sleep is documented in many studies. Chronic alcohol use is associated with longer sleep latency, decreased and disrupted REM sleep, significant alterations in NREM sleep (such as increased NREM sleep or changes in EEG during NREM), and reduced total sleep time [54]. A reduction in REM sleep tends to occur with moderate to large amounts of alcohol [55]. Alcohol withdrawal also affects the amount of REM sleep [54,55]. The results of these studies correspond to ours, and we think that alcohol, by influencing REM sleep, may be a risk factor for SP.

On the other hand, the results of a British study conducted in a group of over 40,000 policemen revealed that officers with bad health habits, i.e., smokers, risky drinking behavior, and absence of physical activity, were characterized by a higher risk of depression and anxiety disorders [25]. Interestingly, in the group of policemen examined by us, alcohol consumption was higher than among nurses and midwives and “other professions”, but this difference was not statistically significant between participants who experienced at least one episode of SP in their lifetime.

The link of SP with the use of psychostimulants in the policemen cohort is disturbing, considering that these substances are associated with the risk of diseases such as hypertension, atherosclerosis, and in this group, we found that a greater number of symptoms of SP were reported [56,57,58]. In the future, it would be worth examining whether, in this profession, the use of the above-mentioned psychostimulants predisposes to other somatic and mental disorders, including other sleep disorders.

Nurses and midwives also represent a professional group exposed to above-average stress and associated with a high risk of mental health problems [25,27,31,59]. In our study, the prevalence rate of SP in this occupational group was 27.9%. This result is significantly higher than the mean of the general population (7.6%) and is close to the prevalence of SP among students (28.3%) and psychiatric patients (31.9%) [10].

The number of SP episodes an individual had during the last month and throughout life was positively correlated with the level of anxiety as a trait measured with the STAI questionnaire. The severity of anxiety as a personality trait was also a factor in the “other professions” cohort, where a positive correlation was found between the number of reported SP symptoms (hallucinations, psychosomatic symptoms, and all symptoms in total), the number of SP episodes in the last year and the number of points in the STAI questionnaire. These results are in line with the results of an earlier study in a group of Polish students, in which we found a positive correlation between the severity of anxiety as a personality trait and the severity of SP symptoms [5]. In kind, a group of Egyptian students who had experienced at least one SP in their lifetime had a higher severity of anxiety as a trait compared to those who had never experienced it [60]. In the context of our observations, the results of Horváth et al. in 2016 are important and interesting. They conducted polysomnographic studies in a group of 1083 patients and noticed that both state anxiety and trait anxiety affect parameters of NREM sleep, while the effect on the parameters of the phase in which SP occurs—REM sleep—is only related to trait anxiety [61]. This finding partially explains our results.

Nurses and midwives and teachers who experienced at least one episode of SP in their lifetime scored higher on the PSS-10 scale compared to those who never experienced it. Moreover, the number of SP episodes during the last month and year in the group of nurses was positively correlated with the intensity of perceived stress on the PSS-10 scale. This scale refers to the stress experienced during the last month. Taking these results into account, it can be hypothesized that the frequency of SP episodes is related to the current perceived stress. Bell et al. also noted a positive correlation between the severity of stress and the number of episodes of SP [62]. The same relationship was observed in a group of Pakistani medical students [63]. Denis et al. pointed to stress as a significant predictor of SP in the multivariate model [64].

The number of psychosomatic symptoms and all SP symptoms reported by the nurses and midwives cohort was positively correlated with the number of points in the PCL-5 questionnaire. A similar relationship was observed in the “other professions” cohort, where the severity of PTSD symptoms also correlated with the number of reported SP symptoms. In the context of these observations, it is worth noting that despite similar relationships in both groups, in the group of nurses and midwives, the intensity of PTSD symptoms in the PCL-5 questionnaire was statistically significantly higher than in the group of “other professions”. However, when comparing only participants who experienced SP, the difference in the number of points in the PCL-5 is not significant. This may suggest that in both SP+ groups, the severity of PTSD symptoms did not differ significantly, and that the severity of PTSD symptoms was associated with a greater severity of episodes of SP. Our observations confirmed the relationship of PTSD with SP, which was indicated in other studies; for example, Jalal and Hinton noticed that students who had experienced at least one SP in their lifetime were characterized by a higher severity of PTSD symptoms [60]. On the other hand, in a study conducted among Pakistani medical students, a positive correlation was found between the severity of PTSD symptoms and the frequency of SP episodes [63]. Another study conducted on a group of Polish students indicated a positive correlation between the number of PTSD symptoms and the severity of psychosomatic symptoms of SP [5]. The study conducted by us in a group of firefighters also confirmed a relationship between PTSD and a higher intensity of SP episodes [11]. In light of the results of our study and the results of other researchers, it can be concluded that there is strong evidence of an association of SP with PTSD. This relationship could be interpreted as two-sided. SP can occur as a result of a traumatic event, and the experience of SP itself can be a traumatic event and cause PTSD [65,66].

The results of our research reveal a relationship between BMI and SP. In the nurses and midwives and “other professions” cohorts surveyed, participants who experienced at least one episode of SP had a lower BMI than those who had never experienced one. In the nurses and midwives cohort, there was a negative correlation between the number of psychosomatic symptoms and all SP symptoms and that of the BMI value. On the other hand, in the group of teachers, we observed an inverse relationship, wherein the number of SP symptoms was positively correlated with the BMI value. Sharpless et al., 2010, noted the positive correlation between the lifetime prevalence of SP and FISP (Fearful Isolated Sleep Paralysis) and the BMI [6]. In other studies, lower BMI was one of the predictors of SP in the multivariate model [11]. In light of these data, the significance of BMI values is unclear in regard to the frequency and severity of SP, and further studies are required. The mechanisms are likely more complex than currently appreciated. It can be assumed that, depending on gender/profession, a higher BMI may either be protective or a risk factor for developing SP. It is worth noting that the average BMI value in the studied groups was around 25, which is the upper limit of the normal BMI.

The presence of SP was also associated with the sleep duration of the respondents. The results varied depending on the profession. Among teachers, sleep duration was negatively correlated with the number of SP symptoms. Policemen who had experienced at least one episode of SP slept for a shorter time on average than those who had never experienced one, and the opposite was observed among nurses and midwives. Moreover, the nurses and midwives cohort slept significantly less than the policemen cohort. The results of previous studies show discrepancies, and it is unclear how sleep duration is related to SP. The authors of two other studies indicate that both too short and too long sleep duration may be a predictor of SP [11,14]. In our opinion, further detailed studies focusing on the individual’s anxiety responsiveness and variables related to sleep architecture in polysomnography and sleep hygiene are required.

The strengths of this study are the relatively large cohorts studied and the extensive number of variables included in the personal questionnaire. The study group consisting of various professions allowed for the comparison of precisely described, unified groups. The online form of the survey provided the respondents with comfort and sufficient time whilst completing the questionnaires.

The main limitation of this study was the form in the online survey, because the online form 1) prevented participants from directly contacting the researcher and asking questions while completing the survey; 2) did not allow us to control the conditions of conducting the survey and 3) did not provide all participants with the same study conditions during the survey. Individuals struggling with SP might have been more motivated to participate in this survey type than others, which could have influenced the estimated prevalence in the studied groups. The tools used in the study focus mainly on the variables selected by researchers, related to the profession, demographics, and lifestyle, as well as PTSD symptoms, perceived stress, and other anxiety symptoms. The study omitted the exploration of sleep quality, possible other sleep disorders, and the presence of symptoms of affective disorders among participants, which is important in terms of sleep paralysis. We also did not assess cognitive functions that may be important in the development of sleep disorders, including sleep paralysis [67,68,69]. In the current study, we focused on a limited number of variables that could affect SP. In the future, it would be worth extending the scope of research tools including the above-mentioned variables. Despite exciting results, some are below RMPE, suggesting that we should interpret the results with caution. Some of them should be not extrapolated to the general population.

The results of our study indicate that the prevalence of sleep paralysis in the occupational groups exposed to stress is higher than in the general population. The presence of this sleep disorder was associated with various variables depending on the professional group studied, which may be used in the future in planning preventive and therapeutic interventions, e.g., the reduction of alcohol consumption and smoking for policemen in particular, while for nurses, focusing on reducing anxiety levels and stress at the outset. Of course, further research is required to detail the impact of the variables we studied, in terms of personality (trait anxiety), stress-related (PTSD), and lifestyle-related variables. It would also be important to define the nature of these relationships because it is not clear whether certain personality traits associated with a greater risk of sleep paralysis choose jobs associated with greater stress or whether difficult working conditions (shift work, high levels of stress) increase the risk of SP. We suppose that the etiology of this disorder is complex and that both factors may be important, interact with each other, and differ between occupations. The third factor, which has not been identified so far, cannot be ruled out either.

## 5. Conclusions

1. The prevalence of SP in each of the researched professional groups was higher than in the general population.
2. The lifetime prevalence of SP was the lowest among policemen (15.5%) and the highest in the group of “other professions” (39.4%).
3. The course and frequency of SP episodes did not differ in individual professional groups.
4. The severity of SP episodes differed between the occupational groups. Hallucination symptoms of SP as well as total symptoms of SP were both more frequently reported among the group of “other professions” as compared to the remaining groups studied.
5. An association of SP with symptoms of PTSD and anxiety was confirmed in the group of nurses and “other professions”.
6. In the policemen cohort, no relationship was found between SP and PTSD and the severity of anxiety symptoms.
7. The frequency and severity of SP, depending on the occupational group, were associated with various lifestyle factors, which may indicate a complex etiology for this sleep disorder.

## Figures and Tables

**Table 1:** Socio-demographic characteristics of participants, by profession group.

Professions:	Nurses and Midwives			Teachers			Policemen			Other Professions		
Sample Type:	Total	SP+	SP−	Total	SP+	SP−	Total	SP+	SP−	Total	SP+	SP−
**% (*n*)**	100 (172)	27.91 (48)	72.09 (124)	100 (107)	26.17 (28)	73.83 (79)	100 (174)	15.52 (27)	84.48 (147)	100 (391)	39.39 (154)	60.61 (237)
**% Female** **(*n*)**	98.84 (170)	100 (48)	98.39 (122)	90.65 (97)	89.29 (25)	91.14 (72)	28.16 (49)	18.52 (5)	29.93 (44)	72.89 (285)	70.13 (108)	74.68 (177)
**% Male** **(*n*)**	1.16 (2)	0 (0)	1.61 (2)	9.35 (10)	0 (0)	8.86 (7)	71.84 (125)	81.48 (22.0)	70.07 (103)	27.11 (106)	29.87 (46)	25.32 (60)
**Age**												
M (RNG)	37.14 (21–63)	34.04 (21–55)	38.33 (22–63)	39.26 (21–62)	36.36 (23–55)	40.29 (21–62)	40.91 (26–59)	39.63 (30–53)	41.14 (26–59)	32.29 (18–67)	29.34 (18–60)	34.21 (18–67)
Me (Range)	36 (42)	30.5 (34)	39 (41)	39 (41)	36.5 (32)	40 (41)	40 (33)	39 (23)	41 (33)	29 (49)	27.5 (42)	32 (49)
**% Place of residence (*n*):**												
City up to 50, 000 inhabitants	19.19 (33)	16.67 (8)	20.16 (25)	26.17 (28)	25.0 (7)	26.58 (21)	28.16 (49)	37.04 (10)	26.53 (39)	19.18 (75)	18.83 (29)	19.41 (46)
City 50,000–100,000 inhabitants	13.95 (24)	12.5 (6)	14.52 (18)	11.21 (12)	3.57 (1)	13.92 (11)	17.24 (30)	29.63 (8)	14.97 (22)	13.55 (53)	12.33 (19)	14.35 (34)
City more than 100,00 inhabitants	46.51 (80)	51.16 (26)	43.55 (54)	37.38 (40)	35.71 (10)	37.97 (30)	37.36 (65)	25.93 (7)	39.46 (58)	42.27 (177)	52.60 (81)	40.51 (96)
% Village	20.35 (35)	16.56 (8)	21.77 (27)	25.23 (27)	35.71 (10)	21.52 (17)	17.24 (30)	7.41 (2)	19.05 (28)	21.99 (86)	16.23 (19)	25.74 (61)
**% Type of work (*n*):**												
-Shift work	81.98 (141)	85.42 (41)	80.65 (100)	7.48 (8)	10.71 (3)	6.33 (5)	58.05 (101)	62.96 (17)	57.14 (84)	24.04 (94)	24.68 (38)	23.63 (56)
-Constant work time	18.02 (31)	14.58 (7)	19.35 (24)	92.52 (99)	89.29 (25)	93.67	41.95 (73)	37.04 (10)	42.86 (63)	75.96 (297)	75.32 (116)	181 (76.37)

Note: Total = all study participants; SP+ = participants with at least one lifetime episode of SP; SP− = individuals who have not experienced SP; M = mean; Me = median; RNG = range.

**Table 2:** Current health status of participants by profession group.

Professions:		Nurses and Midwives			Teachers			Policemen			Other Professions		
Sample Type:		Total	SP+	SP−	Total	SP+	SP−	Total	SP+	SP−	Total	SP+	SP−
**BMI [kg/m^2^]**	M (RNG)	25.37 (17.01–41.52)	24.11 (17.00–40.01)	25.86 (17.26–41.52)	25.11 (17.47–44.44)	25.52 (17.65–44.44)	24.96 (17.47–42.24)	27.15 (17.57–40.04)	27.66 (21.72–35.51)	27.13 (17.57–40.04)	24.73 (15.43–43.16)	24.04 (15.43–51.81)	25.18 (16.81–58.59)
	Me (Range)	24.29 (24.52)	23.05 (23.05)	24.67 (24.26)	24 (26.97)	23.56 (26.8)	24.17 (24.76)	26.59 (22.47)	26.67 (13.79)	26.59 (22.47)	23.46 (43.16)	23.04 (36.38)	23.94 (41.79)
**% Psychiatric Disorder (*n*)**		4.07 (7)	4.17 (2)	4.03 (5)	2.8 (3)	0	3.80 (3)	4.60 (8)	7.41 (2)	4.08 (6)	7.42 (29)	9.09 (14)	6.33 (15)
**% Somatic disorder (*n*)**		45.35 (78)	62.5 (18)	48.39 (60)	35.51 (38)	39.29 (11)	34.18 (27)	26.44 (46)	37.04 (10)	24.49 (36)	27.11 (106)	24.68 (38)	28.69 (68)
**% Medicines taken (*n*)**		43.02 (74)	37.5 (18)	45.16 (56)	31.78 (34)	25 (7)	34.18 (27)	23.56 (41)	29.63 (8)	22.45 (33)	25.06 (98)	22.08 (34)	27.0 (64)

Note: Total = all study participants; SP+ = participants with at least one lifetime episode of SP; SP− = individuals who have not experienced SP; M = mean; Me = median; RNG = range, BMI = body mass index.

**Table 3:** Lifestyle characteristics of participants, by profession group.

Professions:		Nurses and Midwives			Teachers			Policemen			Other Professions		
Sample Type:		Total	SP+	SP−	Total	SP+	SP−	Total	SP+	SP−	Total	SP+	SP−
**Number of cigarettes smoked per day**	M (RNG)	2.87 (0–20)	3.06 (0–20)	2.79 (0–20)	1.13 (0–20)	1.18 (0–12)	1.11 (0–20)	2.97 (0–40)	3.70 (0–40)	2.83 (0–40)	2.48 (0–40)	2.74 (0–20)	2.31 (0–40)
	Me (Range)	0 (20)	0 (20)	0 (20)	0 (20)	0 (12)	0 (20)	9 (40)	0 (40)	0 (40)	0 (40)	0 (20)	0 (40)
**Time of cigarette smoking in years**	M (RNG)	4.4 (0–40)	4.56 (0–35)	4.34 (0–40)	2.02 (0–40)	2.68 (0–28)	1.78 (0–40)	3.67 (0–35)	3.63 (0–30)	3.68 (0–35)	2.64 (0–37)	2.55 (0–28)	2.70 (0–37)
	Me (Range)	0 (40)	0 (35)	0 (40)	0 (40)	0 (28)	0 (40)	0 (35)	0 (30)	0 (35)	0 (37)	0 (28)	0 (37)
**Number of caffe cup per day**	M (RNG)	2.01 (0–8)	1.73 (0–5)	2.12 (0–8)	1.84 (0–5.5)	1.68 (0–5)	1.89 (0–5.5)	1.98 (0–14)	1.2 (0–3)	2.13 (0–14)	1.55 (0–12)	1.48 (0–8)	1.59 (0–12)
	Me (Range)	2 (8)	1.5 (5)	2 (20)	2 (5.5)	1.3 (5)	2 (5.5)	2 (14)	1 (3)	2 (14)	1 (12)	1 (8)	1.5(12)
**Number caffeinated beverages per day**	M (RNG)	0.17 (0–3.5)	0.08 (0–1)	0.21 (0–3.5)	0.19 (0–4)	0.19 (0–2)	0.18 (0–4)	0.22 (0–10)	0.31 (0–2.5)	0.2 (0–10)	0.28 (0–5)	0.38 (0–5)	0.22 (0–5)
	Me (Range)	0 (3.5)	0 (1)	0 (3.5)	0 (4)	0 (2)	0 (4)	0 (10)	0 (2.5)	0 (10)	0 (5)	0 (5)	0 (5)
**The frequency of alcohol consumption per month**	M (RNG)	1.22 (0–4)	1.35 (0–4)	1.16 (0–4)	0.55 (0–1)	1.32 (0–4)	0.65 (0–5)	2 (0–5)	2 (0–4.0)	2 (0–5)	1.58 (0–5)	1.73 (0–5)	1.49 (0–5)
	Me (Range)	1 (4)	0 (4)	0 (4)	1 (1)	0.5 (4)	0 (5)	2 (5)	2 (4)	2 (5)	2 (5)	2 (0–5)	2 (5)
**Average sleep duration per day (h)**	M (RNG)	6.73 (5–11)	7.06 (5–10)	6.6 (5–11)	6.95 (5–9)	6.75 (5–8)	7.03 (5–9)	6.6 (4–9)	6.15 (4–8)	6.68 (4–9)	7.15 (3–12)	7.27 (4–12)	7.08 (3–12)
	Me (Range)	7 (6)	7 (5)	6.5 (6)	7 (4)	7 (3)	7 (4)	7 (5)	6 (4)	7 (5)	7 (9)	7 (8)	7 (9)
**% Satisfaction with the quality of sleep (*n*)**		22.67 (39)	31.25 (15)	19.35 (24)	45.79 (49)	28.57 (8)	51.90 (41)	38.5 (67)	22.22 (6)	41.50 (61)	44.76 (175)	49.35 (76)	41.77 (99)
**% The custom of taking naps during the day (*n*)**		48.84 (84)	47.92 (23)	49.19 (61)	46.73 (50)	53.57 (15)	44.30 (35)	37.93 (66)	44.40 (12)	36.73 (54)	36.83 (144)	41.56 (64)	33.76 (80)
**number of hours of physical activity per week**	M (RNG)	0.73 (0–8)	0.9 (0–5)	0.67 (0–8)	0.79 (0–8)	1.21 (0–8)	0.65 (0–5)	2.03 (0–10)	2.52 (0–8)	1.95 (0–10)	1.51 (0–10)	1.51 (0–10)	1.51 (0–10)
	Me (Range)	0 (8)	0 (5)	0 (8)	10 (8)	0 (8)	0 (5)	1 (10)	3 (8)	1 (10)	0 (10)	0 (10)	0 (10)

Note: Total = all study participants; SP+ = participants with at least one lifetime episode of SP; SP− = individuals who have not experienced SP; M = mean; Me = median; RNG = range; h = hours.

**Table 4:** The number of points in self-report scales among participants by profession group.

Professions:		Nurses and Midwives			Teachers			Policemen			Other Professions		
Sample Type:		Total	SP+	SP−	Total	SP+	SP−	Total	SP+	SP−	Total	SP+	SP−
**The number of points in self-report scales** **Me (Range):**	STAI-T	48 (52)	45 (41)	48 (52)	48(38)	52 (34)	46 (38)	42 (48)	46 (44)	41 (43)	45 (52)	45.5 (51)	45 (51)
	PCL-5	30 (80)	25 (80)	31 (80)	28 (66)	28 (66)	28 (63)	24.5 (78)	23 (52)	25 (78)	23 (71)	21.0 (71)	23 (71)
	PSWQ	57 (64)	52 (56)	57 (64)	60 ((56)	61.5 (44)	59 (55)	46.5 (64)	51 (57)	46 (64)	53 (63)	55 (58)	53 (63)
	PSS-10	21 (36)	20 (31)	21 (36)	21(31)	24 (28)	20 (28)	19 (36)	17 (33)	19 (36)	20 (38)	20 (37)	19 (37)

Note: Total = all study participants; SP+ = participants with at least one lifetime episode of SP; SP− = individuals who have not experienced SP; Me = median.

**Table 5:** Characteristics of SP episodes in the studied SP+ profession groups.

Sample Type	SP+ Nurses and Midwives			SP+ Teachers			SP+ Policemen			SP+ Other Professions		
	**Me**	**M**	**Range**	**Me**	**M**	**Range**	**Me**	**M**	**Range**	**Me**	**M**	**Range**
**Duration of SP episodes (min):**	3	5.49	99	3	4.25	14	1	2.78	9	2.5	5.23	60
**Frequency of SP episodes:**												
-in the last month:	0	0.65	10	1	1.79	18	0	1.04	10	0	0.99	12
-in the last year:	1	5.06	80	3	4.64	50	2	8.74	100	1	7.55	150
-throughout the life:	6	28.80	499	11.5	76	999	12	32.73	299	6	87.14	6999
	**N**	**% (95% CI)**		**N**	**% (95% CI)**		**N**	**% (95% CI)**		**N**	**% (95% CI)**	
**Time of Occurrence of SP episodes:**												
-upon falling asleep	15	31.25 (17.6-44.9)		13	46.43 (26.7-66.1)		11	40.74 (20.9-60.5)		59	38.31 (30.6-46.1)	
-upon awakening	22	44.90 (31.2-60.5)		9	32.14 (13.7-50.6)		9	33.33 (14.3-52.3)		68 27	44.15 (36.2-52.1)	
-both	12	24.49 (10.6-35.3)		6	21.43 (5.2-37.6)		7	25.93 (8.3-43.6)			17.53 (11.5-23.6)	
**Body position during SP episodes:**												
-on back	33	67.35 (55.1–82.3)		19	67.86 (49.4–86.3)		12	44.44 (24.4–64.5)		79	51.30 (43.3–59.3)	
-on stomach	2	4.08 (−1.7–10)		1	3.57 (−3.8–10.9)		2	7.40 (−3.2–18)		9	5.84 (2.1–9.6)	
-sleep position makes no difference	14	28.57 (14.0–40.1)		8	28.57 (10.7–46.4)		13	48.15 (28–68.3)		66	42.86 (35–50.1)	

Note. SP+ = participants with at least one lifetime episode of SP; *N* = number of responders; 95% CI = 95% confidence interval; M = mean; Me = median, min = minutes.

**Table 6:** Prevalence of psychosomatic attributes in ISP experiences in the studied SP+ professions groups.

Sample Type	SP+ Nurses and Midwives		SP+ Teachers		SP+ Policemen		SP+ Other Professions	
	N	% (95% CI)	N	% (95% CI)	N	% (95% CI)	N	% (95% CI)
At least one symptom	47	97.92 (93.7–102.4)	25	89.29 (77.1–101.1)	23	85.19 (70.7–99.5)	146	94.81 (91.3–98.3)
Pressure on chest	31	64.58 (50.6–78.6)	12	42.86 (23.3–62.4)	10	37.04 (17.6–56.5)	77	50 (42.0–57.9)
Unable to breathe	23	47.92 (33.3–62.6)	8	28.57 (10.7–46.4)	9	33.33 (14.3–52.3)	67	43.51 (35.6–51.4)
Chest pain/discomfort	19	39.58 (25.2–53.9)	9	32.14 (13.7–50.6)	7	25.93 (8.3–43.6)	54	35.06 (27.4–42.7)
Feeling of choking	7	14.58 (4.2–24.9)	4	14.29 (0.4–18.5–28.1)	1	3.70 (−3.9–11.3)	9	5.84 (2.1–9.6)
Nausea, abdominal distress	1	2.08 (−2.1–6.3)	0	0	1	3.70 (−3.9–11.3)	5	3.25 (0.4–6.1)
Feeling dizzy, unsteady	18	37.50 (23.3–51.7)	3	10.71 (−1.5–22.9)	5	18.52 (2.9–34.2)	35	22.73 (16.0–29.4)
Sweating	24	50.0 (35.3–64.7)	10	35.71 (16.8–54.6))	15	55.56 (35.5–75.6)	77	50 (42.0–58.0)
Trembling/shaking	19	39.58 (25.2–53.9)	10	35.71 (16.8–54.6)	7	25.93 (8.3–43.6)	55	35.71 (28.1–43.4)
Heart palpitations	40	83.33 (72.4–94.3)	20	71.43 (53.6–89.3)	15	55.56 (35,5–75.6)	107	69.48 (62.1–76.8)
Chills or hot flushes	15	31.25 (17.6–44.9)	8	28.57 (10.7–46.4)	9	33.33 (14.3–52.3)	50	32.47 (24.9–39.9)
Numbness/tingling	30	62.50 (48.3–76.7)	18	64.29 (45.4–83.2)	11	40.74 (20.9–60.5)	87	56.49 (48.6–64.4)
The feeling of body spinning	10	20.83 (8.9–32.7)	2	7.14 (−3.0–17.3)	5	18.52 (2.9–34.2)	42	27.27 (20.2–34.4)

Note. SP+ = participants with at least one lifetime episode of SP; *N* = number of responders; 95% CI = 95% confidence interval.

**Table 7:** The frequency of occurrence of hallucinations during sleep paralysis (SP) in the studied group professions.

Sample Type:	SP+ Nurses and Midwives		SP+ Teachers		SP+ Policemen		SP+ Other Professions	
**Type of Hallucinations:**	N	% (95% CI)	N	% (95% CI)	N	% (95% CI)	N	% (95% CI)
Visual	17	35.42 (21.4–49.5)	8	28.57 (10.7–46.4)	9	33.33 (14.3–52.3)	76	49.35 (41.4–57.3)
Auditory	13	27.08 (14–40.1)	6	21.43 (5.2–37.6)	7	25.93 (8.3–43.6)	55	35.71 (28.1–43.4)
Tactile	11	22.92 (10.6–35.3)	51	35 (20.6–42.9)	51	35 (0.5–29.1)	51	35 (12.6–25.1)
Olfactory	0	0	1	3.57 (−3.8−10.9)	1	3.7 (−3.9–11.3))	2	1.30 (−0.5−3.1)
Intruder	17	35.42 (21.4–49.5)	10	35.71 (16.8−54.6)	10	37.04 (17.6–56.5)	79	51.30 (43.3–59.3)
Incubus	6	12.50 (2.8–22.2)	0	0	3	11.11 (−1.6–23.8)	19	12.34 (7.1–17.6))
Vestibular-motor:	15	31.25 (17.6–44.6)	3	10.71 (−1.5–22.9)	7	25.93 (8.3–43.6)	56	36.36 (28.7–44)
-Feeling of body spinning/flying	10	20.83 (8.9–32.7)	2	7.14 (−3–17.3)	5	18.52 (2.9–34.2)	42	22.27 (20.2–34.4)
-Autoscopy	6	12.50 (2.8–22.2)	2	7.14 (−3–17.3)	4	14.81 (0.5–29.1)	18	11.69 (6.6–16.8)
-OBE	6	12.50 (2.8–22.2)	3	10.71 (−1.5–22.9)	3	11.11 (−1.6–23.8)	29	18.83 (12.6–25.1)
Derealization	7	14.58 (4.2–24.9)	2	7.14 (−3–17.3)	5	18.52 (2.9–34.2)	29	18.83 (12.6–25.1)
depersonalization	6	12.5 (2.8–22.2)	1	3.57 (−3.8–10.9)	3	11.11 (−1.6–23.8)	22	14.29 (8.7–19.9)
Fear	47	97.92 (93.7–102.1)	26	92.86 (82.7–103)	24	88.89 (76.2–101.6)	146	94.81 (91.3–98.3)
Fear of death	27	56.3 (41.7–70.8)	15	53.57 (33.9–73.3)	12	44.44 (24.4–64.5)	86	55.84 (47.9–63.8)

Note. SP+ = participants with at least one lifetime episode of SP; *N* = number of responders; 95% CI = 95% confidence interval.

## Data Availability

The datasets generated and/or analyzed during the current study are not publicly available but are available from the corresponding author on reasonable request.

## Supplementary Materials

The following supporting information can be downloaded at: https://www.mdpi.com/article/10.3390/ijerph19137821/s1, Table S1: List of professions included in the group “Other professions”.
