# Supplementary material for: Characteristics of Sleep Paralysis and Its Association with Anxiety Symptoms, Perceived Stress, PTSD, and Other Variables Related to Lifestyle in Selected High Stress Exposed Professions
Source: Int J Environ Res Public Health. 2022 Jun 25;19(13):7821. doi: 10.3390/ijerph19137821 (PMC9265794; doi:10.3390/ijerph19137821)
Supplement: Supplementary file 1 [file ijerph-19-07821-s001.zip › ijerph-1744810-supplementary.pdf]

Table S1 List of professions included in the group "Other professions"

Profession name (n,%):
office worker (n=84, 21.48%), salesman (n=29, 7.42%), production worker (n=27, 6.91%), hairdresser (n=25, 6.39%), doctor (n=21, 5.37%), psychologist (n=17, 4.35%), professional driver/taxi driver (n=12, 3.07%), manual worker (n=12, 3.07%), farmer (n=11, 2.81%), paramedic (n=11, 2.81%), lawyer (n=9, 2.3%), odd job/no occupation (n=9, 2.3%), entrepreneur (n=8, 2.05%), computer specialist (n=7, 1.79%), profession beautician (n=7, 1.79%), scientist (n=7, 1.79%), construction engineer (n=6, 1.53%), cleaner (n=5, 1.28%), courier (n=5, 1.28%), architect (n=4, 1.02%), babysitter (n=4, 1.02%), call-center worker (n=4, 1.02%), company management (n=4, 1.02%), professional athlete (n=4, 1.02), accountant (n=3, 0.77%), children's animator (n=3, 0.77%), journalist (n=3, 0.77%), pharmacist (n=3, 0.77%), actor (n=2, 0.51%), animal behaviorist (n=2, 0.51%), barista (n=2, 0.51%) carer for the elderly (n=2, 0.51%), commissioner (n=2, 0.51%), cook (n=2, 0.51%), cosmetologist (n=2, 0.51%), English translator (n=2, 0.51%), forester (n=2, 0.51%), musician (n=2, 0.51%), IT worker (n=2, 0.51%), programmer (n=2, 0.51%), receptionist (n=2, 0.51%), biotechnologist (n=1, 0.26%), blogger (n=1, 0.26%), cameraman (n=1, 0.26%), car mechanic (n=1, 0.26%), construction engineer (n=1, 0.26%), dietitian (n=1, 0.26%), electrician (n=1, 0.26%), florist (n=1, 0.26%), graphic designer (n=1, 0.26%), lab technician (n=1, o.26%), librarian (n=1, 0.26%), locksmith (n=1, 0.26%), machine builder (n=1, 0.26%), musician (n=1, 0.26%), nail stylist (n=1, 0.26%), printer (n=1, 0.26%), production operator (n=1, 0.26%), railroad worker (n=1, 0.26%), sport referee (n=1, 0.26%), vet (n=1, 0.26%), waiter (n=1, 0.26%),

Note. n = number of responders, % = percentage of the entire group of "other professions"
